# 盐酸恩沙替尼胶囊的药理与临床评价

**DOI:** 10.3779/j.issn.1009-3419.2020.102.34

**Published:** 2020-08-20

**Authors:** 洋 汪, 晓玢 袁, 佳艳 熊, 志栋 郝, 星哲 彭, 琬琳 陈, 玲玲 崔, 华 李, 秀兰 王, 祥波 何, 敏 杨, 从新 梁, 勇斌 马, 列明 丁, 力 毛

**Affiliations:** 311100 杭州，贝达药业股份有限公司 Betta Pharmaceuticals Co., Ltd, Hangzhou 311100, China

**Keywords:** 恩沙替尼, 肺肿瘤, 间变性淋巴瘤激酶抑制剂, 临床研究, Ensartinib, Lung noplasms, Anaplastic lymphoma kinase tyrosine kinase inhibitor, Clinical studies

## Abstract

肺癌是全世界发病率和致死率最高的恶性肿瘤，其中非小细胞肺癌（non-small cell lung cancer, NSCLC）约占肺癌的85%。间变性淋巴瘤激酶（anaplastic lymphoma kinase, *ALK*）重排阳性的NSCLC仅占全部NSCLC的5%，但预后较差，因此积极的治疗非常迫切。盐酸恩沙替尼胶囊（ensartinib hydrochloride capsule，X-396，商品名贝美纳TM）是第二代ALK抑制剂，对ALK的抑制活性和肺癌中枢神经系统转移的有效性较克唑替尼更强，并且可抑制多个克唑替尼耐药突变位点，临床拟用于治疗克唑替尼耐药的ALK阳性NSCLC。文中对盐酸恩沙替尼胶囊在国内外开展的Ⅰ期-Ⅲ期临床试验进行了总结，并对其药理作用、药代动力学和药效学、临床疗效和安全性评价进行了综述。

## Abstract (en)

Lung cancer is one of the most common malignancies with the highest incidence rate and mortality rate worldwide, and non-small cell lung cancer (NSCLC) accounts for about 85%. Only 5% NSCLC patients are anaplastic lymphoma kinase (*ALK*) rearrangement positive NSCLC, but the prognosis of these patients is poor, and treatment is urgent. Ensartinib (X-396), a next-generation ALK tyrosine kinase inhibitor (ALK-TKI), has shown greater potency on inhibiting ALK activity and controlling brain metastases than crizotinib, which is indicated for the treatment of crizotinib-resistant, ALK-positive NSCLC patients. Several phase Ⅰ to Ⅲ clinical trials included both healthy volunteers and NSCLC patients have been conducted both in China and abroad. In this review, we briefly summarized the results of these trials, and preliminary efficacy, safety, pharmacology and pharmacokinetics/pharmacodynamics of ensartinib were discussed.

肺癌是全世界发病率和致死率最高的恶性肿瘤，占全球恶性肿瘤新发病例的11.6%和恶性肿瘤相关死亡病例的18.4%^[1]^。根据国家癌症中心发布的最新报告^[2]^，2015年我国新发肺癌病例约78.7万人（发病率57.26/10万人），死亡病例约63.1万（死亡率45.87/10万人）。根据组织学和病理类型，肺癌可分为非小细胞肺癌（non-small cell lung cancer, NSCLC）和小细胞肺癌（small cell lung cancer, SCLC）两大类，其中NSCLC约占肺癌的85%，NSCLC患者的5年生存率仅约为10%-15%^[3]^。以铂类药物为基础的化疗或联合治疗，靶向治疗和抗血管生成药物等是不可手术局部晚期或转移性NSCLC的主要治疗方案。常见的肺癌驱动基因包括表皮生长因子受体（epidermal growth factor receptor, *EGFR*）、间变性淋巴瘤激酶（anaplastic lymphoma kinase, *ALK*）、*c-ros*原癌基因1酪氨酸激酶（C-ros oncogene 1 receptor tyrosine kinase, *ROS1*）、细胞间质表皮转化因子（c-mesenchymal-epithelial transition receptor, *c-MET*）等。继表皮生长因子受体酪氨酸激酶抑制剂（EGFR-tyrosine kinase inhibitor, EGFR-TKI）之后，针对*ALK*融合基因为靶点的药物已成为NSCLC靶向治疗领域的焦点。因此，本文针对盐酸恩沙替尼胶囊治疗*ALK*融合基因阳性的NSCLC患者中的药理作用、药代动力学和药效学、临床疗效和安全性评价进行综述。

## 1 *ALK*融合基因及ALK-TKI治疗后的耐药机制

ALK是一种跨膜受体酪氨酸激酶，属于胰岛素受体超家族。在NSCLC中，约3%-7%的患者存在*ALK*基因融合，以ALK酪氨酸激酶区与棘皮动物微管结合蛋白（echinoderm microtubule associated protein-like 4, EML4）5^’^端在2号染色体短臂上发生倒置形成基因融合最为常见。除了*EML4-ALK*之外，与*ALK*基因发生融合的其他基因还包括*TFG*和*KIF5B*等^[4]^。*ALK*阳性多见于不吸烟或轻度吸烟、女性、年轻（年龄 < 50岁）、病理类型为腺癌且不伴有*EGFR*、*ROS1*突变的NSCLC患者。目前常用的检测方法包括荧光原位杂交（fluorescence *in situ* hybridization, FISH）技术、反转录-聚合酶链反应（reverse transcription-polymerase chain reaction, RT-PCR）法、二代测序技术（next-generation sequencing, NGS）和免疫组化（immunohistochemistry, IHC）法^[5]^。第一代ALK-TKI克唑替尼（crizotinib）是美国食品药品监督管理局（Food and Drug Administration, FDA）批准的第一款ALK-TKI，但不可避免的是，多数*ALK*阳性NSCLC患者在接受克唑替尼治疗的1年-2年内会发生耐药，且由于克唑替尼透过血脑屏障的能力较低，临床上发生中枢神经系统（central nervous system, CNS）复发进展的情况也非常多见。克唑替尼的耐药机制主要包括*ALK*继发性耐药突变（37%）和旁路激活。其中，*ALK*继发性耐药突变又可分为*ALK*激酶区二次突变和*ALK*基因扩增。*ALK*激酶区二次突变包括L1196M、L1152R、G1202R、G1269A、1151Tins、S1206Y、C1156Y、F1174C和D1203N等，而针对这种ALK占优势的耐药机制，第二代ALK-TKI对*ALK*融合基因和多种克唑替尼耐药突变均具有更强的抑制作用和高度选择性，并且对中枢神经系统转移瘤也具有较好的疗效。然而二代药物各自的敏感位点不尽相同，塞瑞替尼（Ceritinib）敏感的耐药突变靶点为L1196M、G1269A、S1206Y、I1171T等，对G1202R、F1174C不敏感；阿来替尼（Alectinib）可抑制克唑替尼获得性耐药突变包括L1196M、C1156Y、F1174L、R1275Q等，但对G1202R、V1180L、I1171T等不敏感；布加替尼（Brigatinib）对F1174C、L1196M、S1206R、E1210K、F1245C等耐药位点具有抗肿瘤活性，但对L1198F、S1206C/Y原发耐药^[6]^。虽然三代药物劳拉替尼（Lorlatinib）能克服二代药物共有的G1202R耐药以及血脑穿透力差的问题，但是可能会引起广泛的CNS反应^[7]^，因此，仍有必要开发一款对多个耐药位点敏感且耐受性良好的ALK-TKI，以应对克唑替尼耐药，或者为不能耐受某些二代ALK-TKI药物副作用的患者提供新的选择。

## 2 药理学与毒理学

盐酸恩沙替尼胶囊（ensartinib hydrochloride capsule，X-396，商品名贝美纳^TM^）是由贝达药业有限公司（简称贝达药业）和控股子公司美国Xcovery共同开发新型口服的二代强效的、高选择性ALK-TKI。在分子水平上，对ALK酪氨酸激酶的半数抑制浓度（50% inhibitory concentration, IC_50_）为0.16 nmol/L^[8]^，恩沙替尼对ALK野生型及17个*ALK*突变型的抑制图谱分析结果显示，盐酸恩沙替尼对所有评估的*ALK*突变型均有明显的抑制作用，IC_50_均小于4 nmol/L，其中对野生型*ALK*融合和继发F1174、C1156Y、L1196M、S1206R、T1151等突变位点显示出了强烈的抑制作用，IC_50_均小于0.4 nmol/L；而对G1202突变体的抑制能力则相对较弱，IC_50_值为3.8 nmol/L。除ALK之外，恩沙替尼还对ROS1、原肌球蛋白受体激酶（tropomyosin receptor kinase, TRK）等靶点具有较强的抑制能力（IC_50_ < 1 nmol/L）^[8]^。

细胞水平（*EML4-ALK*融合基因阳性肺癌细胞株H3122）上，恩沙替尼可抑制ALK磷酸化，同时伴有对下游靶点细胞外调节蛋白激酶（extracellular signal-regulated kinase, ERK）和蛋白激酶B（protein kinase B, PKB）的相应抑制。恩沙替尼对肿瘤细胞生长抑制作用的试验结果^[9]^显示，在携带不同ALK融合蛋白的肿瘤细胞，包括H3122肺癌细胞株、H2228肺癌细胞株、SU-DHL-1淋巴瘤细胞株、SY5Y神经母细胞瘤细胞株中，恩沙替尼均具有良好的抗增殖活性，抑制作用是克唑替尼的10倍以上，如在H3122肺癌细胞株中，恩沙替尼和克唑替尼的IC_50_分别为15 nmol/L和180 nmol/L，但恩沙替尼对携带*MET*突变的MKN-45胃癌细胞的生长抑制作用较克唑替尼弱，说明其对ALK的抑制作用和选择性较克唑替尼更高。此外，恩沙替尼对克唑替尼耐药细胞系如Ba/F3-EML4-ALK^L1196M^以及Ba/F3-EML4-ALK^C1156Y^的生长和增殖均显示出一定的抑制作用，其IC_50_分别为106 nmol/L和48 nmol/L，提示盐酸恩沙替尼有克服克唑替尼耐药突变的潜力。脑转移方面，SH-SY5Y小鼠颅内肿瘤试验结果^[9]^表明，盐酸恩沙替尼的颅内药物浓度为65 nmol/L，是抑制EML4-ALK阳性肿瘤生长所需浓度的5倍以上。

恩沙替尼和同类药的作用靶点参见表 1。克唑替尼是一种口服的ATP竞争性小分子抑制剂，其作用靶点为ALK、MET和ROS1^[10, 11]^。与克唑替尼一样，塞瑞替尼是ATP竞争性抑制剂，对ALK的抑制活性是克唑替尼的20倍^[12]^。除以ALK为治疗靶点外，塞瑞替尼对IGF-1R、INSR和STK22D等癌症靶点也有效^[12-14]^。阿来替尼是由罗氏开发的一种高选择性和高效的二代ALK抑制剂，它还具有针对RET的活性^[15, 16]^。布加替尼是ALK/ROS1双靶点抑制剂，对ALK的抑制作用是克唑替尼的12倍^[17, 18]^。劳拉替尼是一种新型小分子ATP竞争性抑制剂，其对克唑替尼耐药患者中所有已知ALK和ROS1基因突变都具有高活性^[19]^。恩沙替尼是ALK、MET、ROS1、AXL的多靶点抑制剂，体外实验证实ALK抑制活性是克唑替尼的3倍-10倍^[9]^。

**1:** 恩沙替尼及同类药的作用靶点 Targets of ensatinib and similar drugs

ALK-TKI	Targets	IC_50_ or Ki ^*^ (nmol/L)	Ref.
Ensartinib	*ALK* *ROS1* *MET* *AXL*	1.7 19 1.8 35	[9]
Crizotinib	*ALK* *ROS1* *MET*	0.69^*^ N.D. 4^*^	[10, 11]
Ceritinib	*ALK* *IGF-1R* *INSR* *STK22D*	0.15 8 7 23	[12-14]
Alectinib	*ALK* *RET*	1.9 4.8	[15, 16]
Brigatinib	*ALK* *ROS1* *FLT3* *EGFR* L858R	0.6 1.9 2.1 1.5	[17, 18]
Lorlatinib	*ALK* *ROS1*	1.3 < 0.02^*^	[19]
ALK-TKI: anaplastic lymphoma kinase-tyrosine kinase inhibitor; IC_50_: 50% inhibitory concentration; EGFR: epidermal growth factor receptor; ^*^Ki and IC_50_ values by *in vitro* enzymatic studies.			

毒理学研究数据显示，在SD大鼠的10 d重复给药毒性试验中，20 mg/kg、40 mg/kg和80 mg/kg剂量组均未观察到与药物相关的毒性反应，因此恩沙替尼的非严重毒性剂量定义为80 mg/kg。在NST剂量下，仅可观察到丙氨酸转氨酶（alanine aminotransferase, ALT）和天门冬氨酸氨基转移酶（aspartate aminotransferase, AST）的升高，幅度小于50%^[9]^。

## 3 药效动力学

在动物模型中，恩沙替尼表现出了很高的抗肿瘤活性，特别是针对ALK驱动的中枢神经系统转移^[9]^。盐酸恩沙替尼经口给药后，能显著抑制*EML4-ALK*阳性人非小细胞肺癌裸鼠和携带*EML4-ALK*^F1174L^耐药突变的人神经母细胞瘤裸鼠体内移植瘤的生长。同等剂量水平下，盐酸恩沙替尼的抑瘤活性显著高于克唑替尼。Paolo等^[20]^研究表明盐酸恩沙替尼可以显著提高携带*EML4-ALK*^F1174L^耐药突变的人神经母细胞瘤裸鼠的平均存活时间，而克唑替尼无此现象；恩沙替尼的颅内浓度是抑制EML4-ALK阳性肿瘤生长所需浓度的5倍以上，而克唑替尼仅为44%，提示盐酸恩沙替尼可以渗透入脑并具有抑瘤活性。

## 4 药代动力学

临床前药代动力学（pharmacokinetics, PK）研究显示，恩沙替尼在体内达峰时间（T_max_）为0.5 h- 4 h，给药后血浆消除半衰期（T_1/2_）为2.8 h-7.8 h。恩沙替尼重复给药体内存在蓄积。药物组织分布试验结果表明，25 mg-100 mg恩沙替尼小鼠灌胃2 h和8 h后，脑内浓度分别为血浆浓度的13%和20%-34%；肿瘤内浓度为血浆浓度的17倍-32倍^[9, 20]^。

现有美国Ⅰ期临床研究中的PK分析结果表明，每日一次口服恩沙替尼50 mg-225 mg后，血浆药物峰浓度（C_max_）和浓度-时间曲线下面积（area under curve, AUC）随剂量增加而增加，而剂量 > 225 mg *qd*的PK参数则呈弱线性关系；多次给药存在蓄积；单次给药12 h后，皮肤中的平均药物浓度是血浆中的9倍左右。在225 mg剂量组，T_1/2_为33.2 h-37.7 h，T_max_为3.1 h-3.6 h，食物对恩沙替尼的吸收无显著影响^[8]^。国内Ⅰ期临床研究中，患者空腹口服恩沙替尼，在150 mg-250 mg剂量范围内，无论单次给药或多次给药，各剂量组平均T_max_为2.67 h-5 h，平均T_1/2_为21.0 h-36.4 h，连续给药约第8-15天体内药物浓度达到稳态^[21]^。PK研究分析提示恩沙替尼*qd*给药方案合理。

## 5 临床研究评价

恩沙替尼已在国内外开展了多项临床研究，涵盖肺癌、晚期实体瘤、非霍奇金淋巴瘤等多个瘤种，现将已开展研究的基本信息总结如下，见表 2。

**2:** 盐酸恩沙替尼胶囊已开展的临床研究 Clinical study progression for ensartinib

Clinicaltrials ID	Study title	Country	Sample size	Primary outcome
NCT01625234	Phase 1/2 study of X-396, an oral alk inhibitor, in patients with ALK-positive NSCLC	U.S.	97	MTD
NCT02959619	Ensartinib in NSCLC patients with positive ALK	China	22	MTD, DLT, RP2D
NCT03510611	A study to investigate the food effect on the pharmacokinetics of ensartinib capsules in Chinese healthy volunteers	China	24	PKparameters, including T_max_, C_max_, AUC_0-t_, AUC_0-∞_, λ_z_, T_1/2_
NCT03536481	Bioequivalency study of ensartinib capsules in healthy volunteers	China	74	PK parameters, including C_max_, AUC_0-t_, AUC_0-∞_, T_max_, T_1/2_, λ_z_
NCT03804541	The absorption, metabolism and excretion of [^14^C]ensartinib in human	China	6	PK parameters, cumulative drug excretion, metabolite identification
NCT03215693	X-396 capsule in patients with ALK-positive NSCLC previously treated with crizotinib	China	160	ORR per RECIST 1.1 based on independent radiology review
NCT03753685	X-396 (Ensartinib) capsules in ALK-positive NSCLC patients with brain metastases	China	27	iORR based on investigator assessment according to RNAO-BM
NCT03213652	Ensartinib in treating patients with relapsed or refractory advanced solid tumors, non-hodgkin lymphoma, or histiocytic disorders with *ALK* or *ROS1* genomic alterations (A pediatric match treatment trial)	U.S.	98	ORR
NCT02767804	eXalt3: study comparing X-396 (Ensartinib) to Crizotinib in ALK positive NSCLC patients	Global	360	PFS assessed by independent radiology review based on RECIST v.1.1
RECIST: Response Evaluation Criteria in Solid Tumors; MTD: maximum tolerated dose; NSCLC: non-small cell lung cancer; DLT: dose limited toxicity; RP2D: recommended phase 2 dose; PK: pharmacokinetic; ORR: objective response rate; iORR: intracranial objective response rate; PFS: progression-free survival.				

### 5.1 Ⅰ期临床研究

恩沙替尼首次人体临床试验（NCT01625234）^[8, 22]^共纳入既往未曾接受ALK-TKI或已接受过多线治疗的97例晚期实体瘤患者，在美国的13家研究中心分别进行剂量递增研究（36例）和扩大入组研究（60例）。剂量递增阶段给予恩沙替尼25 mg-250 mg *qd*治疗，在200 mg *qd*剂量组和250 mg *qd*剂量组分别观察到剂量限制性毒性（dose-limiting toxicity, DLT），表现为3级液体超负荷和3级皮疹各1例；最大耐受剂量（maximum tolerated dose, MTD）未达到。最终Ⅱ期推荐剂量（recommended phase Ⅱ dose, RP2D）定为225 mg *qd*。扩大入组阶段给予恩沙替尼225 mg *qd*持续治疗。基线数据显示，89例（92%）为NSCLC患者，80例（82%）受试者既往接受过至少1种系统性抗肿瘤治疗，包括化疗和ALK-TKI，35例（36%）受试者基线合并存在脑转移。安全性结果显示，盐酸恩沙替尼具有良好的耐受性，86%（83/97）的受试者发生过不良反应，最常见的不良反应为皮疹（56%）、恶心（36%）、瘙痒（28%）、呕吐（26%）和疲劳（22%），程度大部分为1级-2级；3级及以上不良反应的发生率为23%（22/97），主要为皮疹（12%）和瘙痒（5%）；剂量调整或暂停用药的发生率为15%（15/97例）。疗效结果显示，60例疗效可评估*ALK*阳性NSCLC患者中，36例表现为部分缓解（partial response, PR），13例表现为疾病稳定（stable disease, SD），所有剂量组疾病缓解率（objective response rate, ORR）为60%，疾病控制率（disease control rate, DCR）为81.7%，中位无进展生存期（median progression-free survival, mPFS）和中位客观缓解持续时间（median duration of response, mDOR）分别为9.2个月（95%CI: 5.6-11.7）和12.8个月（95%CI: 5.6-24.4）。亚组分析显示，恩沙替尼在初治*ALK*阳性NSCLC患者中的疗效最优，15例既往未经治疗*ALK*阳性NSCLC患者中，ORR为80%，DCR为86.7%，mPFS和mDOR分别为26.2个月（95%CI: 9.2-NR）和24.4个月（95%CI: 7.6-NR）；29例既往仅接受过克唑替尼治疗的患者中，ORR为69%，DCR为96.6%，mPFS和mDOR分别为9个月（95%CI: 5.6-11.7）和7.4个月（95%CI: 3.7-12.9）；16例既往接受过克唑替尼和二代ALK-TKI治疗的患者中，ORR为25%，DCR为50%，mPFS和mDOR分别为1.9个月（95%CI: 1.7-5.7）和4.4个月（95%CI: 0.4-NR）。该Ⅰ期研究提示，恩沙替尼整体上安全性较好，主要不良反应为皮疹且程度较轻，能较好的治疗初治或克唑替尼耐药的*ALK*阳性NSCLC患者。

一项正在国内开展的评价恩沙替尼在中国*ALK*阳性晚期NSCLC患者中安全性、药代动力学特征和初步疗效的Ⅰ期临床研究（NCT02959619）^[21]^共纳入22例患者（剂量递增研究和扩大入组研究各11例）。阶段性研究结果显示，大部分患者为既往接受过标准治疗（包括克唑替尼或塞瑞替尼）后进展的肺腺癌。剂量递增阶段给予恩沙替尼150 mg-250 mg *qd*治疗，150 mg-225 mg剂量组患者均对恩沙替尼展现出良好的耐受性，未出现DLT。在250 mg *qd*剂量组2例患者发生了DLT，均为3级皮疹；因此，仅对225 mg *qd*剂量组进行了扩大入组的临床观察。截止研究结果发表日期，14例疗效可评估ALK阳性NSCLC患者中，9例表现为PR，3例表现为SD，总体ORR为64.3%，DCR为85.7%。安全性方面，所有患者都经历过至少一次不良反应，其中8例发生3级及以上不良反应，以皮疹最为常见（27.3%）。恩沙替尼国内Ⅰ期研究的结果基本与美国Ⅰ期一致，在中国*ALK*阳性NSCLC中表现出显著的疗效和较好的安全性，Ⅱ期临床试验推荐剂量为225 mg *qd*。

此外，恩沙替尼已完成了在健康志愿者开展的评价进食对盐酸恩沙替尼的药代动力学影响的Ⅰ期临床试验（NCT03510611）、在健康志愿者中评价单次口服盐酸恩沙替尼胶囊的随机、开放、两制剂、双周期、双交叉空腹和餐后状态下生物等效性试验（NCT03536481）以及评价[^14^C]恩沙替尼在中国健康男性受试者的吸收、代谢和排泄的单中心、开放标签、单剂量Ⅰ期研究（NCT03804541），研究结果暂未发表。

### 5.2 Ⅱ期临床研究

基于上述Ⅰ期/Ⅱ期临床试验的积极结果，一项在中国开展的单臂、开放性、多中心的Ⅱ期注册性临床研究已于2018年完成入组（NCT03215693），这项研究旨在评价恩沙替尼胶囊治疗克唑替尼耐药的*ALK*阳性NSCLC患者的疗效及安全性^[23]^。合格受试者接受225 mg *qd*的盐酸恩沙替尼胶囊持续治疗，直至疾病进展或满足其他退出标准。研究主要终点是由独立评审委员会评估的ORR。截止至2018年4月11日，该研究已纳入160例既往经克唑替尼治疗疾病进展的Ⅲb期或Ⅳ期ALK阳性NSCLC患者，其中156例受试者进入全分析集（full analysis set, FAS）。FAS分析显示，150例（96%）受试者病理类型为腺癌，151例（97%）患者为肺癌分期为Ⅳ期，除克唑替尼外，85例（54%）受试者既往接受过化疗，55例（35%）接受过放疗，23例（15%）接受过包括EGFR-TKI或抗血管生成药物的其他靶向治疗。疗效结果显示，147例可供独立评审委员会评估疗效的克唑替尼耐药受试者中，ORR为52%，DCR为93%，mPFS为9.6个月（95%CI: 7.4-11.6），中位总生存期（median overall survival, mOS）尚未达到（成熟度17.3%）。安全性方面，145例（91%）受试者发生至少一次不良反应。最常见（发生率≥20%）的不良反应为皮疹（56%）、谷丙转氨酶（alanine aminotransferase, ALT）升高（46%）、谷草转氨酶（aspartate transaminase, AST）升高（41%），程度以1级-2级为主。36例（23%）受试者发生3级不良反应，主要为皮疹（6%）和ALT升高（6%）；未发生4级不良反应。本研究表明恩沙替尼治疗克唑替尼耐药的*ALK*阳性NSCLC疗效显著，且安全性良好。基于此项关键性临床试验的结果，申办方已向国家药品监督管理局药品审批中心提交NDA申请，拟用于治疗既往接受过克唑替尼后进展的或者对克唑替尼不耐受的*ALK*阳性的局部晚期或转移性NSCLC患者。

一项评价恩沙替尼胶囊治疗*ALK*阳性NSCLC伴脑转移患者疗效和安全性的单臂、开放、多中心Ⅱ期临床研究（NCT03753685）正在国内开展，计划入组约27例*ALK*阳性NSCLC伴颅内至少有一处可测量病灶的脑转移患者，主要终点指标为RANO（神经-肿瘤疗效评价）标准确定的颅内有可测量病灶患者的颅内客观缓解率（intracranial objective response rate, iORR）。截止至2019年9月24日，共7例受试者入组并接受恩沙替尼连续给药治疗。同时，在美国开展的评价恩沙替尼治疗复发或难治性晚期实体瘤、非霍奇金淋巴瘤或*ALK*/*ROS1*基因突变的组织细胞疾病的Ⅱ期临床研究（儿科MATCH临床试验，NCT03213652）也在进行当中。

### 5.3 Ⅲ期临床研究

一项随机、开放、国际多中心的Ⅲ期临床研究（eXalt3研究，NCT02767804）^[24]^已于2016年6月启动并开始招募患者，旨在评价恩沙替尼对比克唑替尼应用于晚期*ALK*阳性NSCLC患者一线治疗的有效性和安全性。研究计划入组最多316例患者，截至2018年8月，实际入组290例，主要终点指标为独立评审委员会评估的PFS。目前研究已完成受试者入组，正在随访中。

### 5.4 恩沙替尼对*ALK*阳性NSCLC合并脑转移患者的疗效

在美国开展的恩沙替尼首次人体Ⅰ期/Ⅱ期临床试验（NCT01625234）共纳入29例基线合并脑转移的晚期*ALK*阳性NSCLC患者^[8]^。脑转移患者的疗效结果显示，14例颅内疗效可评估的受试者中，2例达到完全缓解（complete response, CR），7例达到PR，4例为SD，仅1例为PD；患者的iORR为64.3%（9例），颅内DCR（intracranial DCR, iDCR）为92.9%（13例）。其中，9例颅内疾病达到缓解的患者中，3例为ALK-TKI初治，5例既往仅接受过克唑替尼治疗，1例既往接受过克唑替尼和二代ALK-TKI治疗。同时，除2例患者既往接受过全脑放疗（whole brain radiation therapy, WBRT）外，其余7例患者此前均未接受过任何放射治疗。

在中国开展的Ⅱ期注册性临床研究共纳入97例基线合并脑转移的克唑替尼耐药的*ALK*阳性NSCLC患者^[23]^。40例（41%）可供独立评审委员会评估颅内疗效的受试者中，8例既往接受过放射治疗，32例未接受过放射治疗。疗效结果显示，28例受试者疗效达到PR，11例为SD，1例为PD；iORR为70%（28例），iDCR为98%（39例），中位至缓解时间为1.3个月（95%CI: 1.2-1.4），中位颅内缓解持续时间为8.6个月（95%CI: 6.4-NR）。其中，8例之前接受过放射治疗患者的iORR为88%（7例），32例之前未接受过放射治疗患者的iORR为66%（21例）。

在治疗脑转移方面，美国Ⅰ期/Ⅱ期临床研究与国内Ⅱ期注册性临床研究的结果高度一致，均表明恩沙替尼对*ALK*阳性NSCLC合并脑转移患者疗效优越，iORR可达64.3%-70%；其中，恩沙替尼二线治疗克唑替尼耐药患者的iORR为62.5%-70%。塞瑞替尼、阿来替尼和布加替尼二线治疗克唑替尼耐药患者的iORR分别为36%-45%、52%-75%和42%-67%，而克唑替尼一线治疗*ALK*阳性NSCLC的iORR仅为18%-33%^[25, 26]^。因此，尽管暂无头对头的直接比较，从数值上看，恩沙替尼治疗*ALK*阳性NSCLC合并脑转移患者的疗效可能较塞瑞替尼和布加替尼更佳。

### 5.5 恩沙替尼分子标志物和耐药机制研究

基于既往回顾性研究证实了*EML4-ALK*融合亚型对ALK-TKIs敏感性的差异，美国开展的Ⅰ期/Ⅱ期临床研究对基线血浆/组织ctDNA分析的突变位点与恩沙替尼临床疗效之间的关系进行了探索^[8, 9, 27]^。76例基线基因检测分析结果显示，74%的患者存在与疾病相关的突变，最常见的是*EML4-ALK*融合（66%）。15例患者中检测到24处*ALK*激酶结构域突变，最常见的为G1269A（16%），且均为曾接受过ALK-TKI的患者。除此之外，还检测到其他旁路基因突变如*TP53*、*BRAF*、*MET*、*PIK3CA*基因突变及*MET*扩增，发生率最高的为*TP53*突变（35%）。ctDNA突变位点与恩沙替尼临床疗效之间的分析结果显示（*n*=39），恩沙替尼对携带T1151M、L1152V、F1174V、L1196M和G1269A突变位点的患者较为敏感，而对于携带G1202R突变位点的患者敏感性不太稳定。在不同*EML4-ALK*融合亚型中，*EML4-ALK*融合亚型1[V1（*n*=17）：ORR=53%、mPFS=8.2个月]相较于*EML4-ALK*融合亚型3 [V3（*n*=7）：ORR=14%、mPFS=1.9个月]的患者对恩沙替尼更为敏感。携带*TP53*突变患者的无进展生存期较*TP53*基因野生型受试者更短（3.7个月*vs* 11.6个月）。体外敏感性分析结果表明，相比于其他ALK-TKIs，恩沙替尼对L1198F和塞瑞替尼的耐药位点G1123S最为敏感，而对于G1202R和G1269A突变为敏感性较弱。虽然体外研究结果不能完全反映临床疗效，但这也可能解释了虽然部分携带G1202R和G1269A突变的患者对恩沙替尼治疗有响应，但其疗效作用并不持久，且最终会发展成疾病进展。

在中国开展的Ⅱ期注册性临床研究也分析了ALK耐药突变与恩沙替尼疗效的关系^[23]^。探索性研究基线ctDNA分析结果显示：入组的147例可供独立评审委员会评估疗效的克唑替尼耐药受试者中，75例（51%）可检测到*ALK*融合，大部分为*EML4-ALK*（93%），融合类型以V1更常见。45例（31%）受试者携带了*ALK*二次耐药突变，最常见的耐药位点为L1196M、C1156Y、F1174L/V和G1269A；总体ORR为44%，其中F1174L/V（ORR=71%）、G1269A（ORR=67%）、C1156Y（ORR=71%）和T1151（ORR=67%）对恩沙替尼治疗较为敏感。而对于G1202R（ORR=33%）和克唑替尼的耐药位点L1196M（ORR=25%）敏感性相对较弱。

## 6 安全性评价

基于上述国内外临床研究结果，证明了恩沙替尼耐受性良好，安全可控。治疗过程中最常见不良反应主要为皮疹、恶心、瘙痒、呕吐、转氨酶升高和疲劳等，程度大部分为1级-2级，3级及以上不良反应发生率较低，主要表现为皮疹，这些不良反应大部分可以通过减量或停药等措施恢复正常。

## 7 讨论

近年来随着对肿瘤分子机制的认识逐步加深，晚期NSCLC的靶向治疗发展迅速，大大提高了晚期NSCLC患者的生存获益和生活质量。*ALK*阳性NSCLC发生率较低，约占所有NSCLC的3%-7%，且在亚裔和高加索人群之间的发病率差异无统计学意义。既往研究^[28]^报道我国每年新发*ALK*阳性NSCLC病例数接近35, 000例，但根据近期中国一项研究数据显示，在*EGFR*突变阴性的人群中，*ALK*基因融合的发生率可高达12.2%^[29]^。恩沙替尼的临床研究^[27]^显示，*ALK*阳性NSCLC最常见的融合类型为*EML4-ALK*，受试者的年龄为50岁-55岁，多见于不吸烟或轻度吸烟患者，与既往报道高度一致。

克唑替尼是在美国和中国获批的第一款ALK-TKI。PROFILE1014和PROFILE1029临床试验显示克唑替尼一线治疗*ALK*阳性的晚期NSCLC患者的疗效显著优于传统化疗，ORR为74%-87.5%，mPFS为10.9个月-11.1个月，mOS未达到，4年生存率为56.6%^[10, 30, 31]^。克唑替尼显著提高了*ALK*阳性NSCLC患者的临床疗效和生存获益，但由于大部分患者在服用克唑替尼1年-2年后会因发生脑转移或者继发耐药突变而疾病进展，因此可更好地控制颅内疾病或克服耐药突变的多个二代或三代ALK-TKI应运而生。疗效上，恩沙替尼一线治疗*ALK*阳性NSCLC患者的临床疗效与克唑替尼相当或更优，ORR为80%，mPFS为26.2个月，但病例数相对较少（*n*=15）^[8]^。因此，一项比较恩沙替尼与克唑替尼在晚期ALK阳性NSCLC患者一线治疗的有效性和安全性的国际多中心的Ⅲ期临床研究正在开展，计划入组最多316例ALK-TKI初治患者，截止2018年8月，实际入组290例，进一步验证恩沙替尼在一线治疗中的作用。与其他二代ALK-TKI相比，恩沙替尼在治疗克唑替尼耐药患者的疗效明确且突出，ORR为52%，mPFS为9.6个月^[13]^。ASCEND-1和ASCEND-2研究显示，塞瑞替尼二线治疗*ALK*阳性NSCLC的ORR为38.6%-56%，mPFS为5.7个月-6.9个月^[12, 32]^。NP28761和NP28763等研究^[25, 33-35]^显示，阿来替尼治疗克唑替尼耐药的*ALK*阳性NSCLC的ORR为48%-55%，mPFS为8.1个月-8.9个月。布加替尼一项研究（NCT01449461）表明，既往接受克唑替尼治疗耐药患者的ORR为62%，mPFS为13.4个月^[36]^。在2017年世界肺癌大会（World Conference on Lung Cancer, WCLC）大会上公布的ALTA Ⅱ期临床试验结果显示，布加替尼（90 mg维持治疗组或90 mg治疗7 d，随后180 mg维持治疗组）的ORR分别为45%和54%，mPFS分别为9.2个月和12.9个月^[37]^。由于目前缺乏恩沙替尼与其他二代ALK-TKI的随机对照研究，所以直接比较这些同类药物有所限制，但从已有的研究数据看来，与其他二代ALK-TKI的抗肿瘤活性相比，盐酸恩沙替尼的整体疗效和颅内疗效均优于塞瑞替尼，可能与阿来替尼和布加替尼相当。恩沙替尼和同类药治疗*ALK*阳性NSCLC的疗效对比见表 3。

**3:** 恩沙替尼和同类药治疗ALK阳性NSCLC的疗效 Efficacy of Ensatinib and similar drugs in the treatment of *ALK*-positive NSCLC

Drug		ORR	DCR	mPFS (mon)	DOR (mon)	iORR	iDCR
Second-line therapy							
Ensartinib	NCT01625234	69.0%	96.6%	9.0	7.4	62.5%	100.0%
	NCT03215693	52.0%	93.0%	9.6	NA	70.0%	98.0%
Ceritinib	ASCEND-1	56.0%	74.2%	6.9	8.3	36.0%	61.0%
	ASCEND-2	38.6%	77.1%	5.7	9.7	45.0%	80.0%
Alectinib	NP28761	48.0%	80.0%	8.1	13.5	75.0%	89.0%
	NP28673	50.0%	79.0%	8.9	11.2	57.0%	83.0%
	AF-002JG	55.0%	90.0%	NA	NA	52.0%	90.0%
Brigatinib	NCT01449461	62.0%	NA	13.4	NA	50.0%	NA
	ALTA (90 mg)	45.0%	82.0%	9.2	13.8	42.0%	85.0%
	ALTA (90 mg→180 mg)	54.0%	86.0%	12.9	11.1	67.0%	83.0%
Lorlatinib	NCT01970865	39.0%-73.0%	NA	NA	7-NR	46.0%-68.0%	NA
First-line therapy							
Ensartinib	NCT01625234	80.0%	86.7%	26.2	24.4	100.0%	100.0%
Crizotinib	PROFILE 1014/1029	74.0%-87.5%	91.2%	10.9-11.1	11.3	NA	NA
	PROFILE 1005/1007	NA	NA	NA	NA	18.0%-33.0%	56.0%-62.0%
Ceritinib	ASCEND-1	72.0%	89.0%	18.4	17.0	63.0%	63.0%
Alectinib	ALEX	82.9%	88.8%	25.7	NR	85.7%	NA
Brigatinib	ALTA-1L	71.0%	NA	NR	NR	78.0%	NA
DCR: disease control rate; mPFS: median progression-free survival; DOR: duration of response; iDCR: intracranial disease control rate; NA: not available; NR: not reached.							

目前二代或以上ALK-TKI可克服大部分克唑替尼耐药相关性突变，但每个药物的敏感位点不尽相同，如塞瑞替尼敏感的耐药突变位点为L1196M、G1269A、S1206Y、I1171T等；阿来替尼对L1196M、C1156Y、F1174L、R1275Q等位点敏感；布加替尼为F1174C、L1196M、S1206R、E1210K、F1245C等，而三代药物劳拉替尼对以上药物均不敏感的G1202R位点有效。恩沙替尼临床前研究显示对继发F1174、C1156Y、L1196M、S1206R、T1151等*ALK*融合突变位点显示出了强烈的抑制作用。临床研究^[27]^显示，携带了T1151M、L1152V、F1174V、L1196M等突变的受试者对恩沙替尼的治疗较为敏感。在不同*EML4-ALK*融合亚型中，恩沙替尼对V1的效果较V3好，ORR分别为53%和14%，mPFS分别为8.2个月和1.9个月。与其他ALK-TKI相比，恩沙替尼对G1123S和L1198F突变位点的抑制作用最强。虽然临床前研究中恩沙替尼对G1202和G1269A突变体的抑制能力相对较弱，但一些携带G1202的受试者仍对恩沙替尼的治疗有响应。患者服用恩沙替尼耐药后发生在G1269A并突变的频率较高（16%），推测G1269A可能是恩沙替尼的耐药位点之一。

与其他ALK-TKI相比，恩沙替尼安全性更好，且与其他药物的安全性谱也不同。治疗过程中最常见不良反应主要为1级-2级皮疹、转氨酶升高、肌酐升高、恶心、瘙痒、便秘和呕吐等，3级及以上不良反应发生率较低，主要表现为皮疹。关于恩沙替尼引起皮疹的相关机制尚不清楚。但据报道，ALK在正常皮肤的表皮中表达，而且ALK-TKI在体外可抑制正常人表皮角质形成细胞的生长^[38, 39]^。在啮齿类动物中的分布研究^[40]^表明，单次给药后12 h，皮肤中的恩沙替尼浓度比血浆中的浓度高9.0倍，而阿来替尼在皮肤中的含量比血浆高5.7倍，可以初步解释恩沙替尼比其他ALK-TKI更容易出现皮疹的原因。国内Ⅱ期注册性临床研究中，恩沙替尼因不良事件导致剂量调整或中断的发生率分别为12%和15%。同时，仅有5%的受试者发生永久停药。克唑替尼常见不良反应为1级-2级视觉障碍、腹泻、恶心及水肿，3级-4级中性粒细胞减少（11%）及转氨酶升高（14%）也较常见^[10]^。二代ALK-TKI中，塞瑞替尼的最常见的不良反应为ALT升高、AST升高、恶心、腹泻、呕吐、食欲下降和疲劳，发生率均大于30%；3级及以上不良反应主要包括ALT升高（17.1%）、γ-谷氨酰转移酶升高12.1%、疲劳（6.4%）、恶心（6.4%）、腹泻（6.4%）、呼吸困难（5.7%）和AST升高（5%）^[32]^。在ASCEND-4和ASCEND-5的临床研究中，塞瑞替尼因不良事件导致剂量调整或中断的发生率为80%，显著高于恩沙替尼^[41, 42]^。阿来替尼常见的不良反应为1级-2级转氨酶升高、肌酸磷酸激酶升高、疲劳、便秘和水肿，3级及以上肝酶升高等严重不良反应少见^[34]^。布加替尼常见不良反应包括血肌酸磷酸激酶升高、恶心、腹泻、咳嗽及高血压及呼吸困难；3级及以上毒性反应主要包括血压升高（6%）、血肌酸磷酸激酶升高（9%）、肺部相关事件（呼吸困难、缺氧、咳嗽或肺炎，5%）和脂酶升高（3%）^[37]^。高胆固醇血症（59%）、水肿（39%）、周围神经病变（39%）和高甘油三酯血症是（33%）第三代药物劳拉替尼治疗过程中最常见的不良反应，其余包括中枢神经系统影响（如认知、语言、情绪影响）、肝酶升高、视力障碍、便秘、恶心、疲乏等^[43-45]^。恩沙替尼和同类药的常见不良反应参见图 1和图 2。总体上，恩沙替尼安全性良好，3级不良反应主要为皮疹。

**1:**
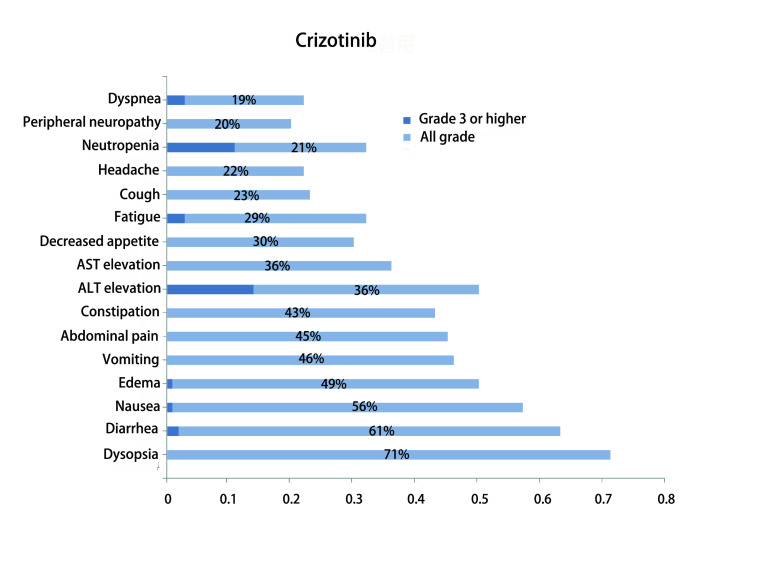
克唑替尼常见不良反应 Common adverse reaction for crizotinib. AST: aspartate aminotransferase; ALT: alanine aminotransferase.

**2:**
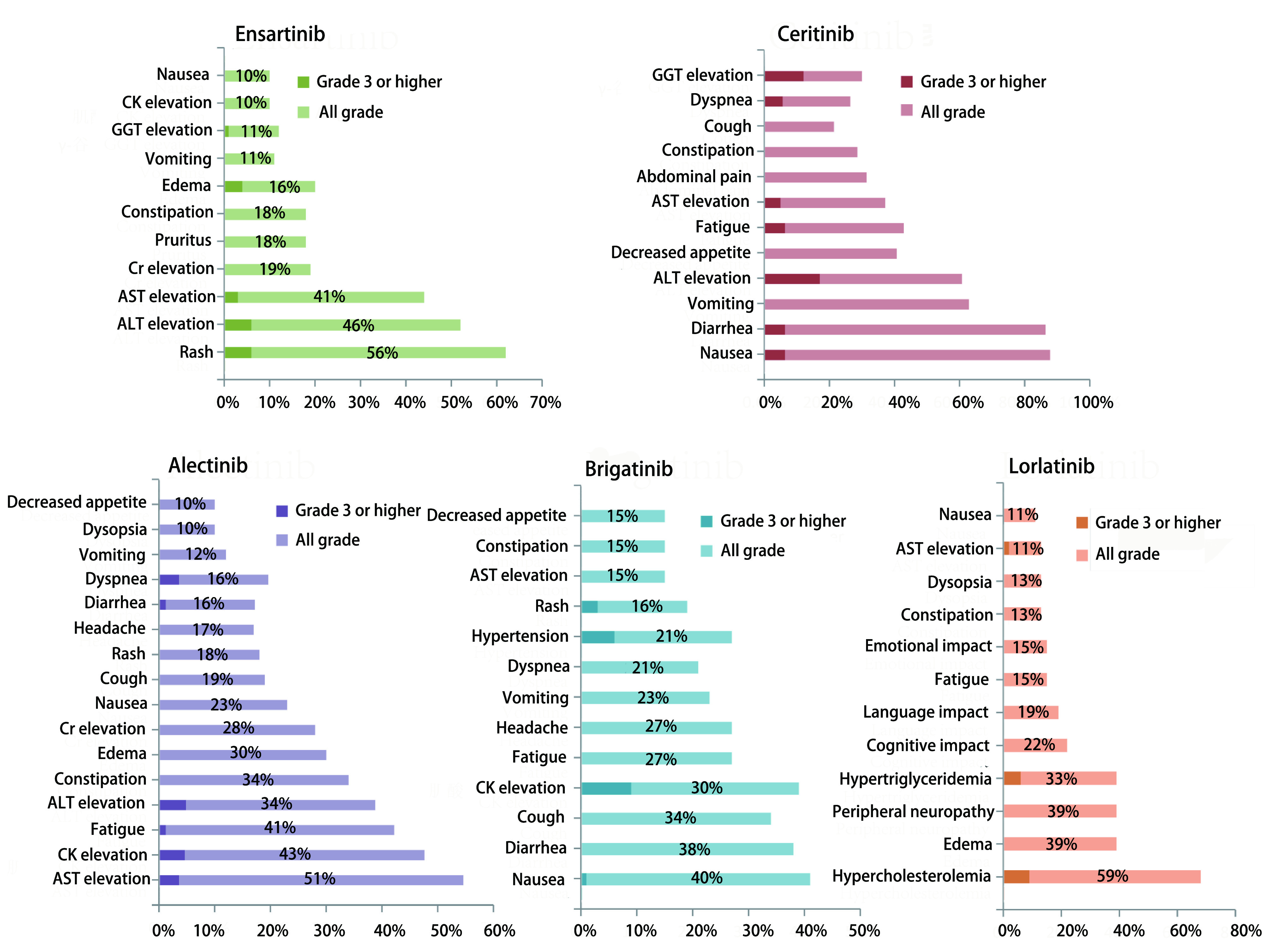
恩沙替尼及其同类药常见不良反应 Common adverse reaction for second- and third-generation ALK inhibitors. Cr: creatinine; CK: creatine kinase; GGT: γ-glutamyl transpeptadase.

## 8 结语

尽管目前已有多款二代及三代ALK-TKI获批，并有多款药物仍在临床阶段，但这些药物对不同的ALK点突变有着不同的敏感度，且具有着不同的毒性特征。随着分子水平的诊断逐渐完善与普及，基于耐药机理的精准用药或个性化用药将越来越受到临床医生青睐。更多的广谱ALK-TKI的研发与上市，将无疑为临床医生在精准用药中提供更多的选择。盐酸恩沙替尼胶囊是我国自主研发、具有自主知识产权、全新分子实体的第二代ALK-TKI，综合临床疗效评价、临床安全性评价以及获益风险评估，盐酸恩沙替尼胶囊具有较好的疗效和安全性，可为广大患者提供新的治疗选择，特别是恩沙替尼对多个克唑替尼及其他二代ALK-TKI的耐药位点均展现出良好的疗效，将进一步扩展ALK阳性NSCLC治疗领域。
